# Anti-PCSK9 antibodies inhibit pro-atherogenic mechanisms in APOE*3Leiden.CETP mice

**DOI:** 10.1038/s41598-019-47242-0

**Published:** 2019-07-31

**Authors:** Susanne Schuster, Sandra Rubil, Matthias Endres, Hans M. G. Princen, Jes-Niels Boeckel, Karsten Winter, Christian Werner, Ulrich Laufs

**Affiliations:** 1 Klinik und Poliklinik für Kardiologie, Universitätsklinikum Leipzig, Leipzig University, Leipzig, Germany; 2 Klinik für Innere Medizin III, Kardiologie, Angiologie und Internistische Intensivmedizin, Universität/Universitätsklinikum des Saarlandes, Homburg, Germany; 3 Department of Neurology with Experimental Neurology, Center for Stroke Research Berlin (CSB), and NeuroCure, Charité University Medicine Berlin, Berlin, Germany; 4 German Center for Cardiovascular Research (DZHK) and German Center for Neurodegenerative Diseases (DZNE), partner site Berlin, Berlin, Germany; 5 TNO-Metabolic Health Research, Gaubius Laboratory, Leiden, The Netherlands; 6 Institute of Anatomy, Medical Faculty, University of Leipzig, Leipzig, Germany

**Keywords:** Cardiology, Cardiovascular biology

## Abstract

LDL-cholesterol (LDL-C) is a causal pathogenic factor in atherosclerosis. Monoclonal anti-proprotein convertase subtilisin/kexin type 9 (PCSK9) neutralizing antibodies are novel potent LDL-lowering drugs which reduce cardiovascular events. To characterize their effect on atherogenesis, APOE*3Leiden.CETP mice were fed a high cholesterol/high fat diet (WTD) or normal chow (NC) for 18 weeks. Mice on WTD were injected with the human anti-PCSK9 antibody mAb1 (PL-45134, 10 mg*kg^−1^ s.c.) or 0.9% saline every 10 days. PCSK9 inhibition decreased total cholesterol in serum of APOE*3Leiden.CETP mice and prevented the development of atherosclerosis. The plaque area in the aortic root was reduced by half and macrophage infiltration determined by Ly6c and Mac-3 staining was ameliorated. PCSK9 inhibition decreased markers of inflammation in mononuclear cells (*Il-6, Tnfa* mRNA), and in serum (CXCL-1,-10,-13; complement factor C5a) compared to control WTD fed animals. The number of circulating Sca-1/VEGF-R2 positive endothelial progenitor cells of the peripheral blood and spleen-derived diLDL/lectin double positive circulating angiogenic cells was increased. To conclude, the PCSK9-mediated anti-atherosclerotic effect involves the upregulation of pro-regeneratory endothelial progenitor cells, a reduction of inflammation and change of plaque composition.

## Introduction

Low-density lipoprotein cholesterol (LDL-C) causally contributes to the pathogenesis of atherosclerosis. Circulating LDL-C is bound by the LDL-receptor (LDLR) which removes it from the circulation by mediating its endocytosis. In 2003, gain-of-function mutations in the gene that encodes serine protease proprotein convertase subtilisin/kexin type 9 (PCSK9) were identified as being associated with autosomal dominant familial hypercholesterolemia. Therefore, PCSK9 became a therapeutic target in lipid lowering therapy and the prevention of CVD^1–4^. PCSK9 gain-of-function mutations are associated with increased levels of LDL-C, while reduced PCSK9 function correlates with lower levels of LDL-C and CVD risk^1,5,6^. Mechanistically, PCSK9 binds to the LDLR directing it to lysosomal degradation^7–9^. By blocking PCSK9, LDLR remain on the cell surface and remove LDL particles from the circulation. In multiple clinical studies, the anti-PCSK9 antibodies alirocumab and evolocumab have been shown to decrease LDL-C level and improve the outcome of CVD^10–15^. While the lipid-lowering effects of PCSK9 inhibitors are well established, knowledge on vascular effects and on the cellular mechanisms that control atherogenesis is still incomplete.

Besides lipid accumulation, atherosclerosis is characterized by chronic inflammation of the arterial wall beginning with the retention of Apolipoprotein B–containing lipoproteins within the subendothelial intima of arteries. This leads to the activation of endothelial cells followed by leukocyte adhesion and migration; foam cell formation and activation of vascular smooth muscle cells leading to increased production of extracellular matrix^16^. Due to reduced effective efferocytosis of apoptotic foam cells, macrophages become necrotic and lead to a thrombogenic and pro-inflammatory environment. Increased cytokine release and proteolytic activity fuel lesion inflammation and plaque instability.

Circulating endothelial progenitor cells (EPC) derived from the bone marrow and are released into the blood to form new blood vessels or to aid in the repair of damaged vessels^17–20^. After recruitment to sites of ischaemia and endothelial damage, EPC integrate into the endothelium and positively regulate endothelial cell growth and neovascularization^19,21,22^. Inflammation, metabolic and vascular risk factors and type 2 diabetes have been shown to downregulate EPC numbers and impair their function^22–26^. In contrast, statin-mediated lipid lowering or physical activity improve EPC numbers and cell function^27–29^. In a large prospective clinical study, CD34/KDR-positive cells were shown to predict cardiovascular events^18^. Within the EPC population, circulating angiogenic cells (CAC) can be distinguished^19^. CAC derive from myeloid haematopoietic cells and share features with monocytes and exert their angiogenic effects via paracrine and signaling mechanisms.

Here, we aimed to characterize the vascular effects of PCSK9 inhibition in APOE*3Leiden.cholesteryl ester transfer protein (CETP) mice, a mouse model for familial dysbetalipoproteinemia with human-like lipoprotein metabolism^30–32^, on atherosclerosis development, inflammation and EPC and CAC number.

## Results

### Anti-PCSK9 treatment decreases the size of aortic atherosclerotic plaques and pro-inflammatory macrophage infiltration

**Figure 1:**
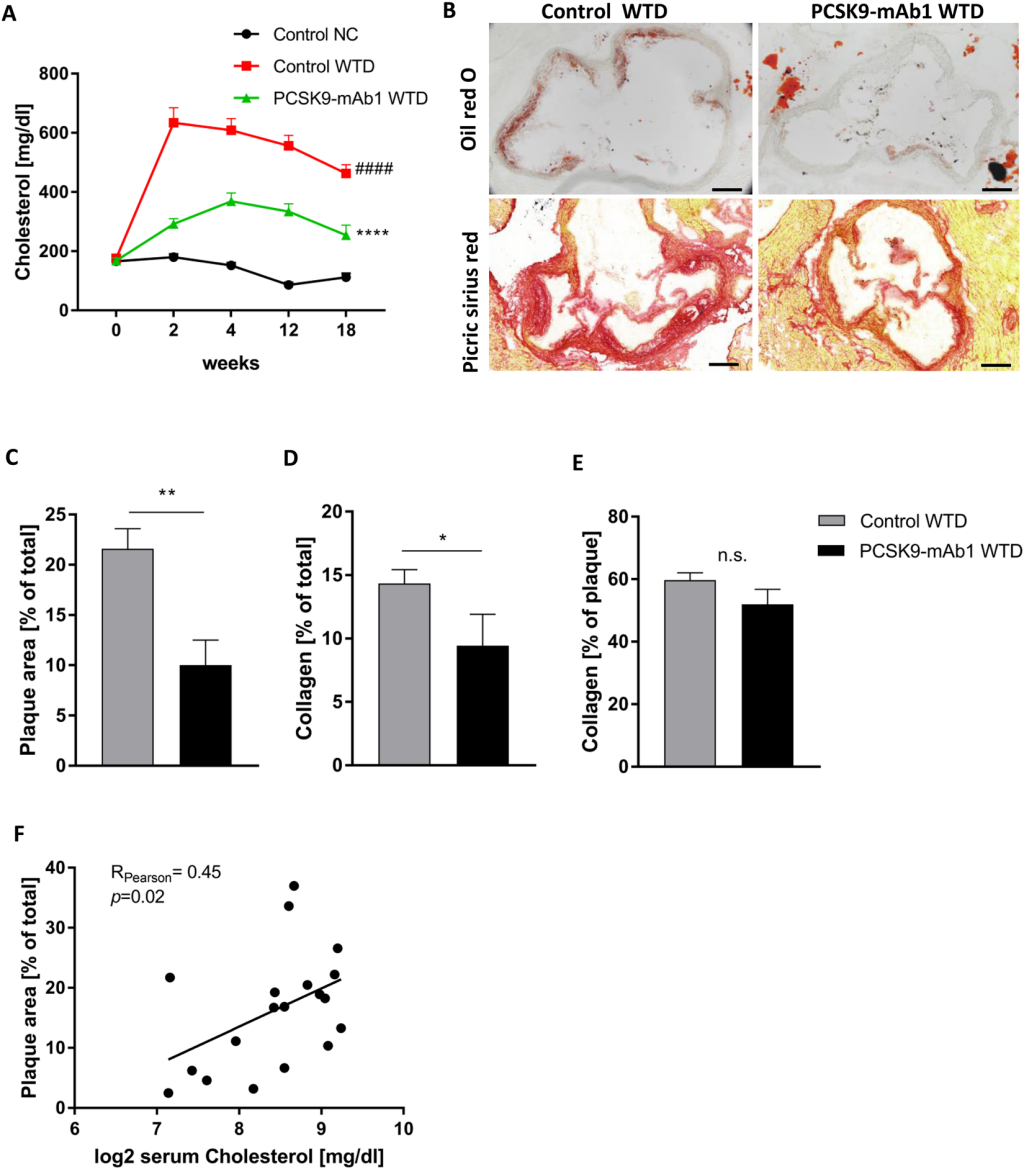
Anti-PCSK9 antibody treatment decreases total cholesterol in serum and the size of aortic atherosclerotic plaques of APOE*3Leiden CETP mice. (**A**) Serum cholesterol levels are presented as group means ± SEM (Control normal chow (NC) n = 6, WTD n = 12, PCSK9-mAb1 n = 10). Statistical comparison was performed using two-way ANOVA and Sidak’s multiple comparison post-hoc test. ^####^p < 0.0001 versus control NC, ****p < 0.0001 versus control WTD (Western-type diet). (**B**) Representative aortic root sections with oil red O staining and picric sirius red staining of atherosclerotic plaques (10 × magnification, bar 200 um) after 18 weeks of anti-PCSK9 antibody (PCSK9-mAb1) treatment are shown. (**C**) Histomorphometric quantification of atherosclerotic plaques after 18 weeks anti-PCSK9 antibody treatment, shown as percentage of plaque area [% of total sinus area]. **p < 0.01 versus control WTD group (WTD n = 10, PCSK9-mAb1 n = 9). (**D**) Picric sirius red staining analysis of collagen content in aortic sinus after 18 weeks anti-PCSK9 antibody treatment referred to total aortic sinus area or (**E**) to total plaque area. n.s. (not significant). Data are presented as group means ± SEM (WTD n = 8, PCSK9-mAb1 n = 6). Statistical comparison was performed using unpaired Student’s t-test. (**F**) Correlation analysis of log2-transformed serum cholesterol level and atherosclerotic plaque area [% of total] was performed (Pearson correlation R = 0.45, *p* = 0.02).

APOE*3Leiden CETP mice fed a Western-type diet (WTD) were injected with the anti-PCSK9 antibody mAb1 (PL-45134, PCSK9-mAb1) every 10 days for 18 weeks^33^. Cholesterol levels were measured after 2, 4, 12 and 18 weeks and were compared to APOE*3Leiden CETP mice treated with 0.9% saline as control. PCSK9 inhibition decreased total cholesterol in the serum of APOE*3Leiden CETP mice (baseline: control + normal chow (NC) 166 ± 41 mg/dl, control + WTD 176 ± 48 mg/dl, PCSK9-mAb1 + WTD 168 ± 38 mg/dl; 18 weeks: control + NC 112 ± 32 mg/dl, control + WTD 463 ± 103 mg/dl, PCSK9-mAb1 + WTD 254 ± 108 mg/dl, *p* < 0.0001) (Fig. 1A). Histomorphometric analysis of atherosclerotic lesions in the aortic sinus showed that mice treated with the PCSK9 antibody have reduced atherogenesis. The plaque area was reduced by half compared to saline-treated mice (control + WTD 22 ± 3%, PCSK9-mAb1 + WTD 10 ± 3%, *p* < 0.01) (Fig. 1B,C). Collagen content was normalized to the total aortic sinus area (Fig. 1D) and to the total plaque area (Fig. 1E). PCSK9 inhibition reduced the amount of collagen in the total aortic sinus area by 34% from 14 ± 1.1 (% of total area) in control + WTD mice to 9 ± 2.5 (% of total area) in PCSK9-mAb1 + WTD mice (*p* < 0.05) (Fig. 1D). The amount of collagen in atherosclerotic plaques was not changed (control + WTD 60 ± 2% vs. 52 ± 5% in PCSK9-mAb1 + WTD, not significant) (Fig. 1E). Correlation analyses revealed a significant positive association between serum cholesterol level and plaque size (R_Pearson_ = 0.45, *p* = 0.02) (Fig. 1F).

**Figure 2:**
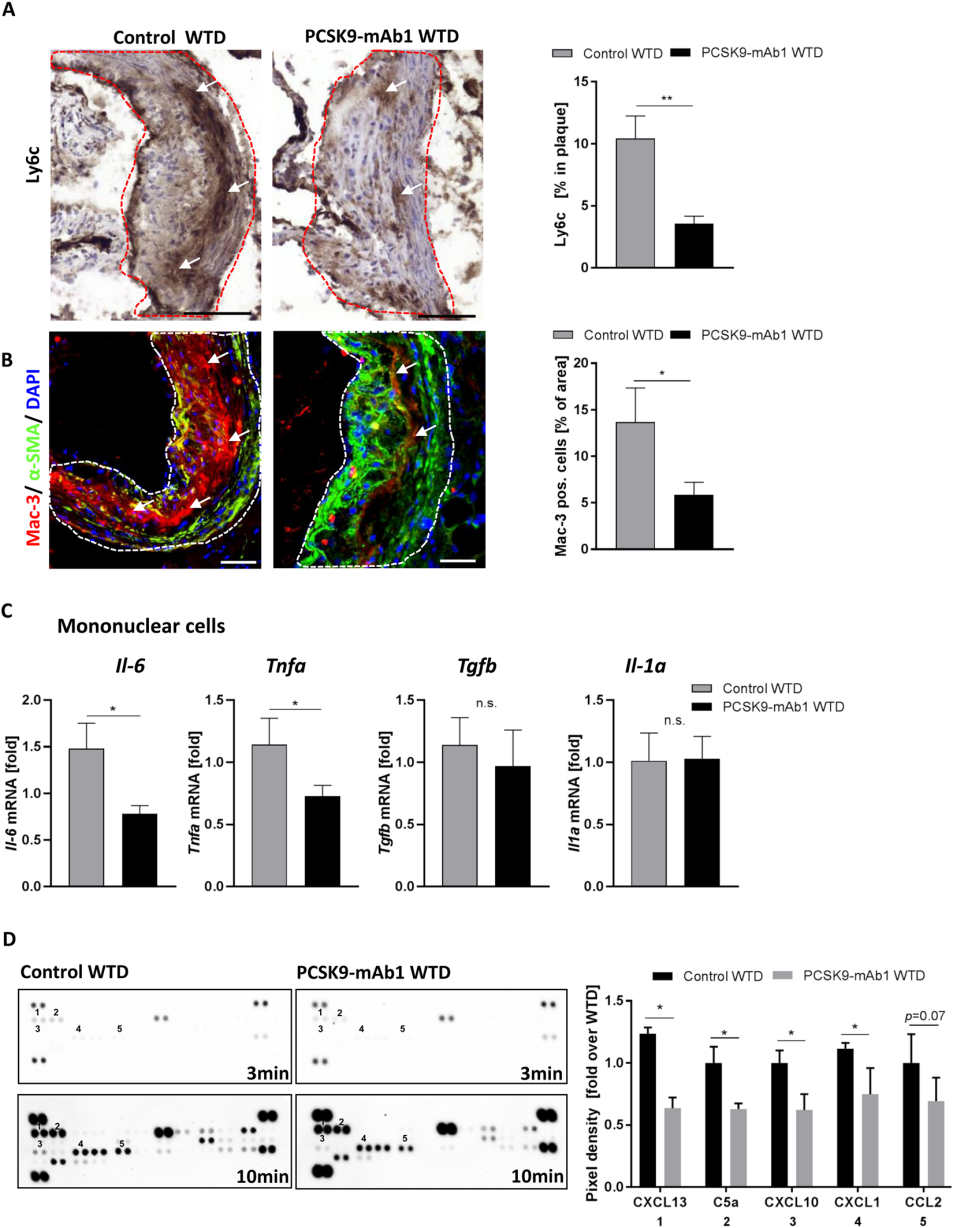
Anti-PCSK9 antibody treatment reduces pro-inflammatory macrophage infiltration and downregulates inflammation in APOE*3Leiden CETP mice. (**A**) Representative immunostaining for Ly6c in frozen aortic root sections and histomorphometric quantification of Ly6c positive cells in aortic plaques (shown as % of area in aortic plaque) (20 x magnification, bar 100 um) (WTD n = 5, PCSK9-mAb1 n = 12). (**B**) Representative immunofluorescent Mac-3 (red) and α-smooth muscle cell (α-SMA) (green) staining of frozen aortic root sections and quantification of positive cells in aortic plaques (depicted as % of area in atherosclerotic plaque). Nuclei were stained with DAPI (blue) (WTD n = 6, PCSK9-mAb1 n = 5) (20 x magnification, bar 100 um). (**C**) Real-time PCR analysis of *Il-6*, *Tnfa*, *Tgfb* and *Il-1a* mRNA expression in spleen-derived MNC of APOE*3Leiden.CETP mice on a WTD and treated with anti-PCSK9 antibody (PCSK9- mAb1, n = 9) or saline (n = 10) every 10 days for 18 weeks. (**C**) Representative membranes (3 min and 10 min exposure) of mouse serum cytokine array of WTD (n = 4) and PCSK9-mAb1 (n = 4) treated mice. Pixel density was quantitated by ImageJ and results are depicted as fold over WTD. Data are presented as mean ± SEM and fold over control WTD which was set 1. Unpaired Student’s t-test was performed. *p < 0.05, ***p < 0.001 versus control WTD (Western-type diet).

Pro-inflammatory macrophages and chemotactic cytokines are involved in atherosclerotic disease progression. Macrophage accumulation in the intima promotes lesion development and fuel atherosclerosis progression^16^. To analyze pro-inflammatory macrophage infiltration in the carotid arteries, immunostainings of Ly6c and Mac-3 were performed. Ly6c is expressed in mononuclear phagocytes and is upregulated during macrophage differentiation^34^. As depicted in Fig. 2A,B, PCSK9 inhibition ameliorated macrophage infiltration in the atherosclerotic plaque. WTD-fed control mice showed pro-inflammatory macrophage infiltration, while mice injected with the anti- PCSK9 antibody exhibited markedly reduced infiltrated macrophages (Ly6C: control + WTD 10 ± 1.8% vs. 4 ± 0.6% in PCSK9-mAb1 + WTD, *p* < 0.01; Mac-3: control + WTD 14 ± 3.7% vs. 6 ± 1.3% in PCSK9-mAb1 + WTD, *p* < 0.05). To determine the effect of PCSK9 inhibition on pro-inflammatory gene expression, mRNA expression of *Il-6, Tnfa, Tgfb* and *Il-1a* were analyzed in isolated mononuclear cells (MNCs). The pro-atherosclerotic cytokine genes *Il-6* and *Tnfa* decreased by 41.0 ± 6.2% and by 27.2 ± 8.6% (*p* < 0.05), respectively, in PCSK9-mAb1 treated mice compared to control + WTD animals (Fig. 2C). The expression levels of *Tgfb* and *Il-1a* were not changed. Serum analysis revealed that chemokines, such as CXCL-1, -10, and -13 as well as the complement factor C5a were downregulated in PCSK9 antibody treated mice compared to WTD mice (Fig. 2D). Taken together, these data show an anti-inflammatory effect of LDL-cholesterol lowering by PCSK9 inhibition.

### Administration of anti-PCSK9 antibody increases circulating endothelial progenitor cells and angiogenic cells

**Figure 3:**
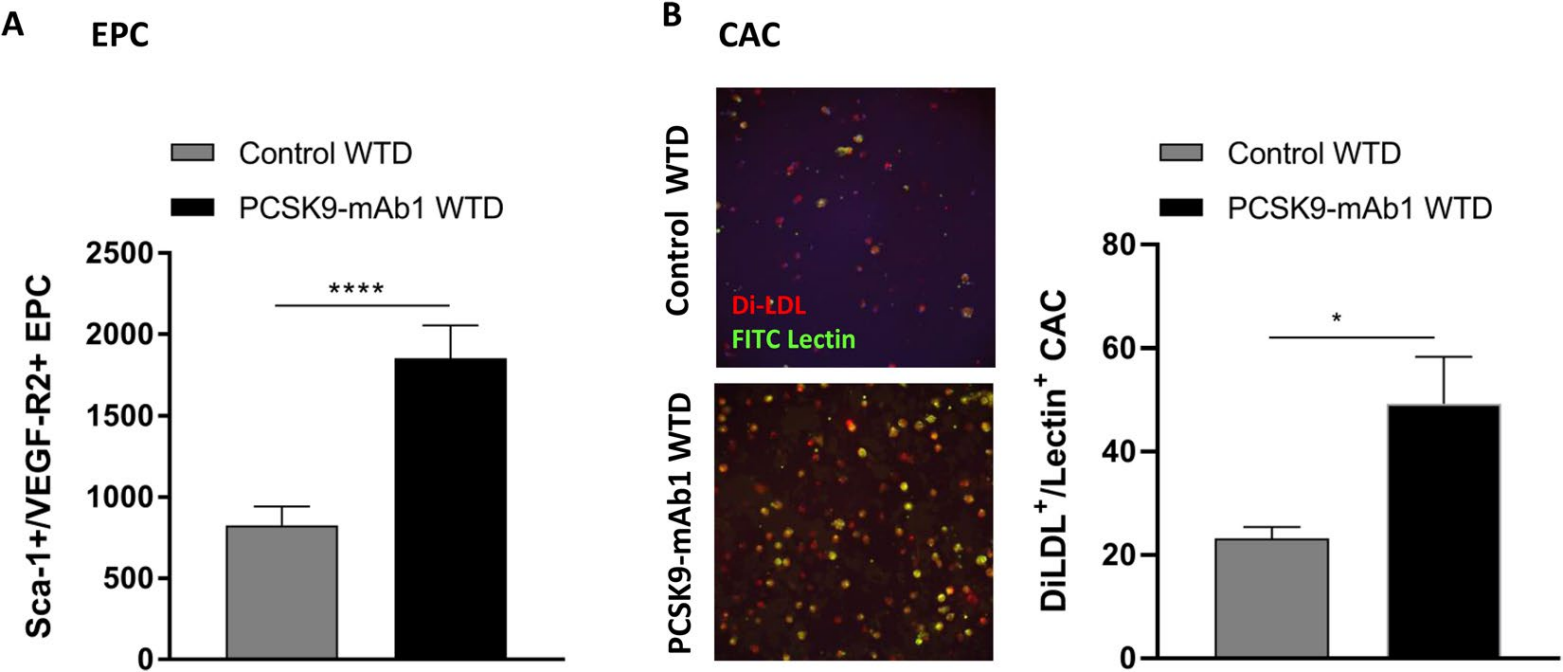
Anti-PCSK9 antibody treatment enhances number of circulating endothelial progenitor cells (EPC) and angiogenic cells (CAC) in APOE*3Leiden CETP mice. (**A**) Effects of PCSK9 inhibition in APOE*3Leiden.CETP mice compared with controls after 18 weeks on WTD on the number of Sca-1/VEGF-R2 positive EPC in the blood per 25,000 gated events as measured by FACS analyses (WTD n = 6, PCSK9-mAb1 n = 5). (**B**) Representative picture of spleen-derived diLDL/lectin positive CAC after treatment with anti-PCSK9 antibody (PCSK9- mAb1 WTD, n = 5) or saline (Control WTD, n = 6) every 10 days for 18 weeks. Only double positive cells for red diLDL and green lectin staining were considered CAC and included in the analysis. Data are presented as mean ± SEM and unpaired Student’s t-test was performed. *p < 0.05 versus control WTD (Western-type diet).

Sca-1/VEGF-R2 positive EPC regulate endothelial homoeostasis, function and new vessel formation^21,22,29,35^. The EPC number in peripheral blood increased by 2.3- fold after 18 weeks (Sca-1 + /VEGF-R2 + per 25.000 lymphocytes: control + WTD 827 ± 115, PCSK9-mAb1 + WTD 1856 ± 200, *p* < 0.0001) (Fig. 3A). Further, *in vitro* culture of spleen-derived MNC was used to differentiate circulating angiogenic cells (CAC). WTD fed mice treated with anti-PCSK9 antibody showed increased numbers of CAC compared to control mice (cells per microscopic field: control + WTD 23.2 ± 2.7, PCSK9-mAb1 + WTD 49.3 ± 5.1, *p* < 0.05) (Fig. 3B).

## Discussion

The present study demonstrated that the treatment with the anti-PCSK9 antibody mAb1 reduced atherosclerosis development, macrophage infiltration and cardiovascular inflammation and increased the number of EPC and CAC in APOE*3Leiden.CETP mice fed a Western type diet.

In line with previous studies, we found that inhibition of PCSK9 reduces serum cholesterol levels and further attenuates atherogenesis and plaque inflammation^13,36–39^. Studies in Pcsk9 knock-out mice showed increased hepatic LDLR levels that were linked to reduced circulating LDL cholesterol^40^. The association between serum cholesterol level and atherosclerotic plaque size observed in the present study indicates a causal link of serum cholesterol and plaque formation^39^. Furthermore, there is a strong association between the severity of coronary atherosclerosis and adverse cardiac events^41,42^. The prevention of atherogenesis in anti-PCSK9 antibody treated mice was accompanied by reduced collagen deposition. This is in accordance with findings of Nicholls *et al*. who found no alterations in fibrous components in atheroma after evolocumab therapy in coronary artery disease patients^41^. However, an *in vivo* study with alirocumab has shown improved lesion composition in atherosclerotic mice by increasing collagen content^39^.

In addition to the reduction in plaque lipid load, the present study demonstrated that PCSK9 inhibition also affects plaque composition by reducing the amount of activated monocyte-derived macrophages in the atheroma. This suggests that the LDL-C lowering effect by PCSK9 inhibition may also lead to reduction in monocyte transmigration through the arterial wall as well as to reduced differentiation into mononuclear phagocytes. Those phagocytes can transform into cholesterol-laden “foam cells” as they ingest the accumulated normal and modified lipoproteins. Alirocumab therapy was further shown to ameliorate monocyte adhesion and trafficking as well as lesion composition in APOE*3Leiden CETP mice^39^. This is in line with our findings that PCSK9 inhibition downregulates chemokine levels, such as CXCL-1, -10, -13, which can be expressed by activated endothelial cells, smooth muscle cells as well as emigrated leukocytes. They are key players involved in the recruitment and infiltration of immune cells into the vessel wall and exacerbate atherosclerosis progression^43,44^. Besides ameliorated chemokine levels C5a is reduced in PCSK9 antibody treated mice. C5a is one of the most potent inflammatory chemoattractants^45^. It activates endothelial cells and regulates the secretion of various cytokines and chemokines such as Tnfa, IL-6 and CCL-2 -12,-13^46^.

Noteworthy, monoclonal antibodies only target PCSK9 in the circulation, whereas the pathophysiological relevance of intracellular PCSK9, such as in intra-atheroma macrophages, requires better understanding^47^. Further, our study revealed the novel observation that LDL-C lowering with anti-PCSK9-antibody significantly increased the number of circulating EPC and CAC that are considered markers of endothelial and vascular health and are associated with positive clinical outcomes^18^. Additionally, PCSK9 expression is positively associated with apoptosis in vascular endothelial cells, tumor cells, and neurons^48,49^ suggesting a detrimental role for PCSK9 on endothelial repair and vasculogenesis. Indeed, Chao *et al*.^50^ reported that PCSK9 serum levels correlated with apoptosis in circulating endothelial cells which may provide the explanation for our observation.

In conclusion, our data show that PCSK9 inhibition prevents the development of atherosclerotic plaques. This effect is mediated by several anti-atherogenic mechanisms that involve the up-regulation of pro-regenerative endothelial progenitor cells, reduction of inflammation in circulating cells, in the serum and in the plaque as well as changes of the plaque composition. These data provide important mechanistic explanations for the observed reduction of clinical outcomes with the human monoclonal antibodies against PCSK9 that have been reported in the meantime^13,14^.

## Methods

### Animals and anti-PCSK9 antibody treatment

APOE*3Leiden.CETP transgenic mice were fed *ad libitum* a western type diet (WTD: 21% fat, 19.5% casein, 1.25% cholesterol) or normal chow (NC) (Ssniff, Soest, Germany) for a period of 18 weeks. Mice on WTD were injected with the human anti-PCSK9 antibody mAb1 (PL-45134, 10 mg*kg^−1^ subcutanously (s.c.), n = 10) or 0.9% saline (control, n = 12) every 10 days for 18 weeks. The antibody was generated as described previously^33,36^ and provided by Amgen. Mice were euthanized with ketamine and xylazine^51^. The experiments were approved by the *Universität des Saarlandes* and complied with national guidelines (directive 63/2010 of the European Parliament) as well as the ARRIVE guidelines for reporting experiments involving animals^52^.

### Serum cholesterol quantification

Venous blood was obtained before and after treatment with anti-PCSK9 antibody mAb1 or 0.9% saline. Cholesterol concentration was measured in serum using the standard curve based LabAssay™ Cholesterol Kit (Wako, Neuss, Germany) according to the manufacturer’s instructions.

### Staining of frozen aortic root sections

Atherosclerotic plaques and collagen deposition in the aortic root was quantified as described previously^51^. Atherosclerotic plaque area is expressed as % of total aortic sinus area. Picric sirius red staining (0.1%) was used to analyze collagen deposition in total aortic sinus as well as in atherosclerotic plaque area. Pro-inflammatory macrophage infiltration into the arterial wall was determined by immunostaining of frozen cryosections of the aortic root with Ly6c antibody (1:100, Cat.ab15627) (Abcam, Cambridge, UK) and Mac-3 antibody (CD-107b, Clone M3/84, 1:50,Cat 550292, BD, Franklin Lakes, USA) and corresponding secondary ImmPRESS® anti-rat HRP conjugated antibody (Vector Laboratories, Burlingame, CA, USA) or anti-rat Alexa 594 antibody (Dianova, Hamburg, Germany), respectively. For immunofluorescent α-SMA staining, the primary antibody from Abcam (1:250, Cat.ab5694, Abcam, Cambridge, UK) and the corresponding anti-rabbit Alexa Fluor 647 conjugated secondary antibody (Invitrogen, Carlsbad, USA) were used. Stained sections were fully digitalized at 20 × magnification using a digital slide scanner (Pannoramic Scan II, 3D HISTECH Ltd., Budapest, Hungary). Images of stained tissue slices were captured from slide scanner data sets (Pannoramic Viewer, version 1.15.4., 3D HISTECH Ltd., Budapest, Hungary) in TIFF format at 10 × or 20 × magnification and were quantitated by ImageJ using the plugin color deconvolution as described in^53^. Percentage (%) of area was calculated from grey-scaled pictures.

### Quantification of CAC

Mononuclear cells were isolated from spleen by Ficoll density gradient centrifugation as described previously^51^. CAC were identified by plating 4 × 10^6^ MNC on fibronectin-coated 24-well plates and by the uptake of DiLDL (1,1′-dioctadecyl-3,3,3′,3′- tetramethylindocarbocyanine-labelled acetylated LDL, 2.4 μg·mL^−1^; CellSystems, St. Katharinen, Germany) and binding to FITC-labelled Ulex europaeus agglutinin I (lectin, 10 μg·mL^−1^; Sigma-Aldrich). DAPI was used for nuclei staining. To quantitate CAC, diLDL-lectin-double positive CAC were counted in four random fields and normalized to total cell number using a Nikon Eclipse E600 fluorescence microscope and NIS 3.0 BR software (Nikon, Düsseldorf, Germany).

### Quantification of EPC by flow cytometry

Mouse blood was used to characterize and quantitate EPC by flow cytometry as described previously^51^. Mouse equivalent surface markers Sca-1 (FITC conjugated), VEGFR2 (PE-conjugated)^20,54^ and appropriate isotype controls (IgG2a κ FITC and PE, BD Pharmingen) were used. FACS analysis was performed on a FACS Calibur instrument (BD) and Cell Quest software version 6.0 (BD Biosciences, Heidelberg, Germany).

### Gene expression analysis

Quantitative PCR assays to detect *Il-6*, *Tnfa*, *Tgfb* and *Il-1a* mRNA expression were performed in MNC from mice after Ficoll isolation of spleen homogenates (*Il-6* forward: 5′- tcc tac ccc aat ttc caa tg -3′; *Il-6* reverse: 5′- acc aca gtg agg aat gtc ca -3′; *Tnfa* forward: 5′- acg gca tgg atc tca aag ac -3′; *Tnfa* reverse: 5′- aga tag caa atc ggc tga cg -3′; *Tgfb* forward: 5′- agc ccg aag cgg act act at -3′; *Tgfb* reverse: 5′- tcc aca tgt tgc tcc aca ct -3′; *Il-1a* forward: 5′- ctc tag agc acc atg ctac aga c -3′; *Il-1a* reverse: 5′- tgg aat cca ggg gaa aca ctg-3′). PCR data were calculated using the comparative CT Method (ΔΔCT Method) and were normalized on *18 S* (*18 S* forward 5′- tca aca cgg gaa acc tca c -3′, *18 S* reverse 5′- acc aga caa atc gct cca c -3′) as housekeeping gene.

### Cytokine array

To analyse serum cytokines and chemokines, we used the membrane-based Proteom Profiler Array™ (R&D, Minneapolis, USA) and followed manufacturer’s instruction. Multiple exposure times were applied (1–10 min) and pixel density was quantitated by ImageJ after background substraction and normalization to positive control on the membrane. Data are expressed as fold over WTD fed mice which was set at 1.

### Statistical analysis

Statistical analyses were performed with Graph Pad Prism (version 7; Graph Pad Software Inc., La Jolla, CA, USA). Unless otherwise stated, all data are presented as mean ± SEM. For analysis, Student’s two-tailed, unpaired t-test (for parametric data), Mann-Whitney test (for non-parametric data) and ANOVA for multiple comparisons were employed where applicable. Post-hoc comparisons were performed with the Neuman–Keuls test or Sidak’s multiple comparison test. P-values of p < 0.05 were considered significant.
